# Deep learning-based rapid generation of broadly reactive antibodies against SARS-CoV-2 and its Omicron variant

**DOI:** 10.1038/s41422-022-00727-6

**Published:** 2022-09-27

**Authors:** Hantao Lou, Jianqing Zheng, Xiaohang (Leo) Fang, Zhu Liang, Meihan Zhang, Yu Chen, Chunmei Wang, Xuetao Cao

**Affiliations:** 1 Frontier Research Center for Cell Response, Nankai-Oxford International Advanced Research Institute, College of Life Sciences, Nankai University, Tianjin, China; 2 Ludwig Institute for Cancer Research, University of Oxford, Oxford, UK; 3 The Kennedy Institute of Rheumatology, University of Oxford, Oxford, UK; 4 Big Data Institute, University of Oxford, Oxford, UK; 5 Department of Engineering Science, University of Oxford, Oxford, UK; 6 Target Discovery Institute, Centre for Medicines Discovery, Nuffield Department of Medicine, University of Oxford, Oxford, UK; 7 Chinese Academy for Medical Sciences Oxford Institute, Nuffield Department of Medicine, University of Oxford, Oxford, UK; 8 Department of Immunology, Centre for Immunotherapy, Institute of Basic Medical Sciences, Chinese Academy of Medical Sciences, Beijing, China

**Keywords:** Innate immunity, Bioinformatics

Dear Editor,

The COVID-19 pandemic has been ongoing for nearly two and half years, and new variants of concern (VOCs) of SARS-CoV-2 continue to emerge, which urges the development of broadly neutralizing antibodies.^1,2^ Variants such as the delta (B.1.617.2 lineage) and Omicron (BA.1 and BA.2) were reported to exhibit immune evasion to some of the current therapeutic antibodies.^2,3^

The ever-evolving SARS-CoV-2 calls for rapid prediction of antibody binding to new variants and development of broadly neutralizing antibodies. Considering the application of deep learning in antibody engineering and optimization, we wonder whether the broadly reactive antibodies against SARS-CoV-2 variants can be rapidly designed and generated by deep learning. Here we report the development of an Atrous Convolution Neural Network (ACNN)^4^ based deep learning framework: cross-reactive B cell receptor network (XBCR-net) that can predict broadly reactive antibodies against SARS-CoV-2 and VOCs directly from single-cell BCR sequences. XBCR-net composes of two parts, the first part extracts the features relevant to the antibody–antigen interaction via three-branch ACNN, and the second part predicts the binding probability of the antibodies to antigens (14 different RBD sequences) by a residual structural Multi-Layer Perceptron (Fig. 1a; Supplementary information, Fig. S1a). The performance of the ACNN-based XBCR-net prediction on SARS-CoV-2 binding was evaluated, showing significantly higher accuracy, precision and recall value than other frameworks (Fig. 1a; Supplementary information, Fig. S1b).

**Fig. 1:**
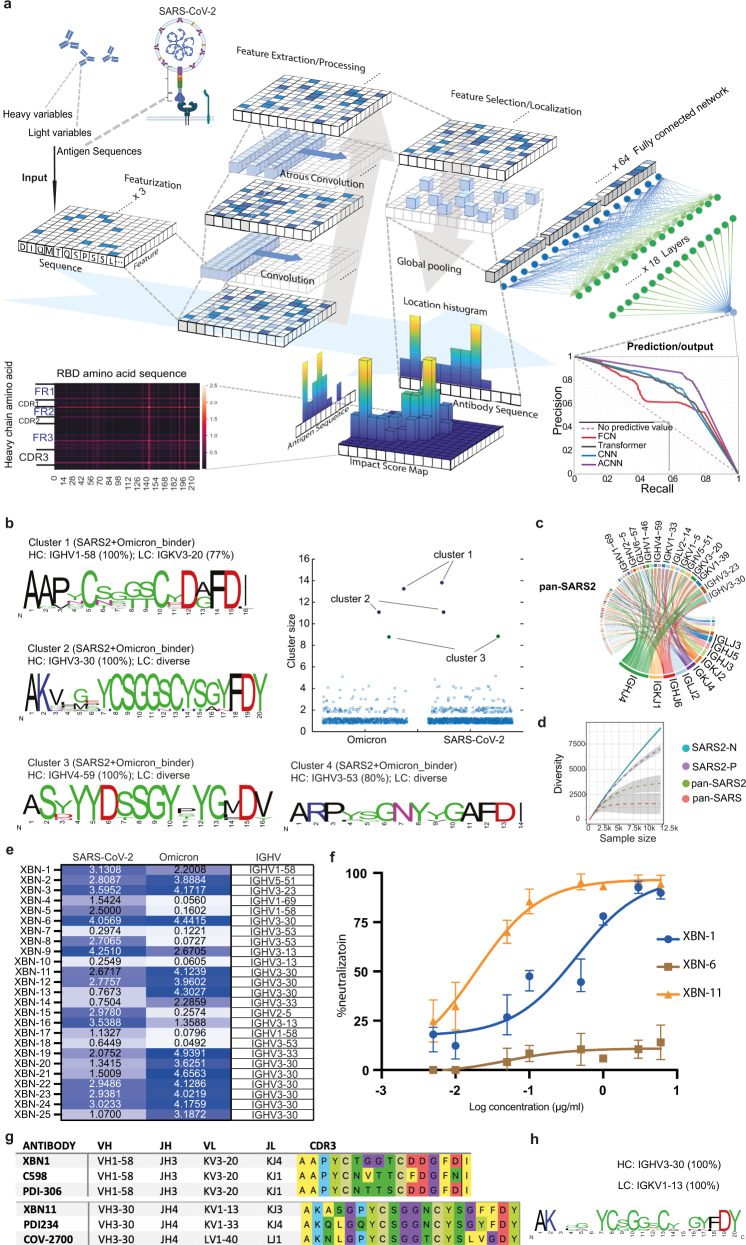
Development of broadly reactive antibodies by ACNN-based deep learning framework XBCR-net. **a** The features of the amino acid sequences of VH, VL and RBD sequences were extracted, localized and max-pooled to be concatenated together as input to the fully connected layers. The active features in the latent space were then processed by Multi-Layer Perceptron to predict the binding probability of antibody to multiple antigens. The impact score of VH, VL and RBD is calculated on the local histogram impact score map, representing how much weight is given to the specified amino acids on VH, VL (*y* axis) and RBD (*x* axis). Prediction results are evaluated by Precision-Recall curves of ACNN (Violet), Transformer (Gray), FCN (Red) and CNN (Blue). **b** The HCDR3 sequences of the predicted SARS-CoV-2 and Omicron variant binders are clustered by using an 80% sequence similarity. Cluster size represents the number of BCR sequences in the cluster. For each expanded cluster, the HCDR3 sequences are visualized as a sequence logo plot, where *y*-axis represents the frequency of the individual amino acid at the corresponding position in *x*-axis. The frequency of the dominating VH gene is listed above the logo. **c** Circos plot showing the frequency of antibodies encoded by the specified V region to J region pairing of the pan-SARS2 sequences. **d** The diversity of the four groups of BCR repertoire is analyzed, which is linked to the sample number of each group. **e** Binding of the predicted cross-reactive antibodies to RBD of SARS-CoV-2 and Omicron variants (left panel) was examined by ELISA. Representative OD reading is plotted as heatmap ranging from 0.05 to 5.0, and OD of 0.1 is used as cut-off value (*n* = 3 per group). **f** The SARS-CoV-2 Omicron variant (BA.1) pseudovirus neutralization curves of XBN-1, XBN-6 and XBN-11 mAbs were generated from luciferase readings at 8 dilutions (*n* = 3). **g** HCDR3 sequences of the XBN-1 and XBN-11 are aligned with the most convergent anti-SARS-CoV-2 antibodies from the published studies. **h** The HCDR3 sequence frequency of the dominant cluster (encoded by IGHV3-30 and IGKV1-13) of the pan-SARS group is shown.

To evaluate the adaptability of XBCR-net to unseen VOCs, RBD of the new Omicron variant (BA.1, BA.2 and BA.4) and 142 anti-Omicron mAbs (including therapeutic antibodies LY-CoV016, AZD-1061, REGN10933 and S309) were used for testing.^5^ XBCR-net predicted 102 out of the 142 binders as positive and 116 out of the 142 non-binders (anti-SARS-CoV-2 antibodies that do not bind Omicron) as negative, illustrating the practicality of XBCR-net in predicting Omicron binding antibodies without prior knowledge (Supplementary information, Table S1, Data S3).

We then used XBCR-net to predict wild-type (WT) SARS-CoV-2 and VOC binders from a single-cell BCR dataset of the COVID patients (GSE171703), who are not infected by the Omicron variant.^6^ We identified 153 and 89 clusters based on 80% HCDR3 sequence similarity from predicted SARS-CoV-2 binders and Omicron variant binders (Fig. 1b). Three clusters have a size greater than 8 and are predicted to be cross-reactive to both SARS-CoV-2 and Omicron variants. The dominant cluster (cluster 1) is highly convergent to a well-studied public clonotype encoded by IGHV1-58, including the therapeutic antibody Tixagevimab, which is reported to neutralize SARS-CoV-2.^7^ Two other clusters (clusters 2 and 3) also belong to the public anti-SARS-CoV-2 clonotypes encoded by IGHV3-30 (such as therapeutic antibody REGN 10987) and IGHV4-59 V-region (such as cross-reactive antibody 47D11), respectively (Fig. 1b). The cluster 4 antibodies were also described in some studies, such as COV2-2733 and COV2-2752, which bind to SARS-CoV-2 but not SARS-CoV.^8^

XBCR-net predicted that 336 out of 6743 BCRs were cross-reactive to the RBD region of the WT SARS-CoV-2 and its VOCs (pan-SARS2, including alpha, beta, delta and gamma variants), while only 54 of them showed cross-reactivity towards RBD of SARS-CoV (pan-SARS). The V–J region usage of the pan-SARS-2 showed slightly higher IGHV3-30, IGHV3-23 and IGKV1-39 gene usage (Fig. 1c). More biased usage of IGHV3-30 and lower diversity were observed in the sequences of the pan-SARS compared with the pan-SARS2 antibody sequence repertoire (Fig. 1d; Supplementary information, Fig. S2a, b).

Because of the biased IGHV3-30 usage and enlarged IGHV3-30 cluster that we observed in the predicted cross-reactive RBD binders (Fig. 1c; Supplementary information, Fig. S2a–c), we selected 10 IGHV3-30 antibodies and 15 antibodies with various IGHV usage from the filtered antibody lists (described in Supplementary information). All 25 mAbs showed significant binding to RBD of WT SARS-CoV-2 compared with negative control antibodies at 1 μg/mL. In agreement with the Omicron validation dataset, 20 of 25 mAbs were also cross-reactive to RBD of the SARS-CoV-2 Omicron variant at 1 μg/mL (Fig. 1e). Interestingly, all the IGHV3-30 antibodies in our study were able to bind Omicron variant (Fig. 1e). To further empirically validate the XBCR-net, we applied it to the cloned 25 mAbs for SARS-CoV binding. Out of the 8 mAbs predicted to cross-react to SARS-CoV, 6 of them bound significantly to the RBD of SARS-CoV (Supplementary information, Fig. S3). These results demonstrated the capability of XBCR-net in extrapolating the BCR cross-reactivity to emerging variants without additional training data.

SARS-CoV-2 Omicron variants have been reported to evade the neutralization by some therapeutic mAb drugs. We then tested the neutralization competence of these cross-reactive mAbs on the Omicron and delta variants of SARS-CoV-2. XBN-1 showed neutralization activity against both delta (B.1.617.2) and Omicron (BA.1) with IC_50_ of 7 ng/mL and 418 ng/mL, respectively. XBN-6 neutralized delta (D614G) with IC_50_ of 1200 ng/mL while XBN-11 displayed neutralization to Omicron (BA.1) with IC_50_ at 17 ng/mL (Fig. 1f; Supplementary information, Table S2).

Because SARS-CoV-2 is continuously evolving, treatments of the new variants need to be updated rapidly for clinical decisions.^9,10^ Our XBCR-net can predict antibody binding to the newly discovered variants of SARS-CoV-2 rapidly after acquiring the RBD sequences. The IGHV1-58 mAb we cloned showed convergence to the published antibodies PDI-306 and C598 which neutralize SARS-CoV-2. The IGHV3-30 mAb we cloned showed heavy chain convergence to published coronavirus antibodies PDI234 and COV2-2700 (Fig. 1g). The PDI234 and COV2-2700 do not bind to SARS-CoV,^8^ indicating that key mutations on the HCDR3 and different light chain (IGKV1-13) can render the IGHV3-30 clonotype mAbs cross-reactive. From the prediction of XBCR-net, we found mAbs derived from the cluster encoded by IGHV3-30 and IGKV1-13 can bind to SARS-CoV and Omicron variant in addition to SARS-CoV-2 (Fig. 1e, h; Supplementary information, Fig. S3), suggesting that the IGHV3-30, IGKV1-13 encoded cluster we identified can be further developed to be broadly neutralizing antibodies against SARS-CoV and SARS-CoV-2. In sum, our XBCR-net can predict the broadly reactive antibodies against newly discovered variants of SARS-CoV-2 without prior knowledge of new variant-specific antibodies, contributing to the rapid generation of antibodies against SARS-CoV-2 variants and other emerging viruses.

## Supplementary information

Supplementary information

Supplementary information, Data S1

Supplementary information, Data S2

Supplementary information, Data S3

## Data Availability

The neural network models have been uploaded to github https://github.com/jianqingzheng/XBCR-net.
