# Supplementary material for: Deep learning-based rapid generation of broadly reactive antibodies against SARS-CoV-2 and its Omicron variant
Source: Cell Res. 2022 Sep 27;33(1):80–2. doi: 10.1038/s41422-022-00727-6 (PMC9514701; doi:10.1038/s41422-022-00727-6)
Supplement: Supplementary file 1 — Supplementary information [file 41422_2022_727_MOESM1_ESM.pdf]

Supplementary information

Materials and Methods

Neural network

The network is based on two part and the ACNN architecture was described as in previous studies [1,2]. In the first part of our neural network, amino acid sequences of a paired variable region of antibody heavy and light chains (VH and VL), as well as the sequences of the receptor-binding domain (RBD) of SARS-CoV, SARS-CoV-2 and its VOCs (excluding omicron variants) plus eight other coronaviruses, are fed to a three-branch weight-sharing ACNN[1] (**Fig. 1a**). The pairs of antigens and antibodies sequences are input to the networks and processed with four steps to estimate the probability of binding, including featurization, feature extraction, feature selection and classification.

For XBCR-net training, a total of 3265 antibodies against the RBD of SARS-CoV-2 WT, alpha, beta, delta, gamma variants, SARS-CoV and the other 8 coronavirus were inputted as the ground truth for positive binding (collected from CoV-AbDab database)[3], while another 2071 anti-coronavirus antibodies that do not bind the RBD region were used as the negative training set (**Supplementary information, Data S1**). In addition, 5066 anti-HIV and 6378 anti-HCV antibodies were used as an extra negative input for training (collected from Observed Antibody Space), assuming they do not have cross-reactivity against coronavirus.

The dataset (of antibody-antigen interaction data points) was divided into two folders, 90% of them for network training and the other 10% for performance validation. The performances of XBCR-net using ACNN, Transformer, convolutional neural network (CNN) and fully connected network (FCN) were compared to select the best

framework for the first part. We found that ACNN outperforms the other two networks with significantly higher accuracy and recall (**Supplementary information, Tables S3, S4**). The more detailed evaluation of the Precision Recall Curve (PRC) and Receiver Operating Characteristics (ROC) curve also reveals the significant advantage of ACNN for the better trade-off between the Precision and Recall rate (**Fig. 1a; Supplementary information, Fig. S1b**).

In featurization step, each amino acid on the chain of heavy, light and antigens are encoded into a one-hot vector denoted as $v_{\mathrm{h}}, v_{\mathrm{l}}, v_{\mathrm{a}} \in \{0,1\}^{20}$, and the amino acids are then concatenated to three sequences' matrices (feature maps) $s_{\mathrm{h}} \coloneqq [v_{\mathrm{h}1} \cdots v_{\mathrm{h}t}], s_{\mathrm{l}} \coloneqq [v_{\mathrm{l}1} \cdots v_{\mathrm{l}t}], s_{\mathrm{a}} \coloneqq [v_{\mathrm{a}1} \cdots v_{\mathrm{a}t}] \in \{0,1\}^{20\times t}$, where t denotes the standardized length of input encoded sequences with zero-padding.

In feature extraction step, three-branch weight sharing ACNN is used to extract the intra-sequences feature vectors and processes as feature maps in a latent space:

$$f_{\mathrm{h}} = \mathrm{ACNN}(s_{\mathrm{h}})$$

$$f_{\mathrm{l}} = \mathrm{ACNN}(s_{\mathrm{l}})$$

$$f_{\mathrm{a}} = \mathrm{ACNN}(s_{\mathrm{a}})$$

Where $f_{\mathrm{h}}, f_{\mathrm{l}}, f_{\mathrm{a}} \in \mathbb{R}^{c\times t}$ denote the processed feature maps, c denotes the feature channel number.

In feature selection step, the feature maps are filtered to select the features $\hat{f}_{\mathrm{h}}, \hat{f}_{\mathrm{l}}, \hat{f}_{\mathrm{a}} \in \mathbb{R}^{t}$ via global max-pooling from each feature channel dimension:

$$\hat{f}_{\mathrm{h}} = \mathrm{pool}(f_{\mathrm{h}})$$

$$\hat{f}_{\mathrm{l}} = \mathrm{pool}(f_{\mathrm{l}})$$

$$\hat{f}_{\mathrm{a}} = \mathrm{pool}(f_{\mathrm{a}})$$

Finally, the selected features are concatenated and fed into a residual structural MLP to process the inter-sequence information and predict a probability:

$$p = \text{MLP}([\hat{f}_\text{h} \ \hat{f}_\text{l} \ \hat{f}_\text{a}])$$

Where $p \in [0,1] \cap \mathbb{R}$ is the output from a sigmoid activation function at the end layer of MLP to estimate the binding probability.

Compared with CNN, ACNN utilizes a series of atrous convolution layers, with the atrous rates computationally optimized to extend the receptive field without any losses of resolution resulting from down-sampling. Thus, Atrous-III block is able to achieve broader field of view with higher resolution than Res-conv block, providing more comprehensive and informative features (**Supplementary information, Fig. S4**). Thus, ACNN is able to achieve broader field of view with higher resolution than CNN, providing more comprehensive and informative features. Transformer employs attention mechanism for a global-range receptive field, but also requires the positional encoding to preserve the locational information and a larger dataset to fulfil its potential. A 5-folder cross validation is used to compare the three modules as recorded in (Supplementary information, Table S3). Because the sequence length of heavy, light and antigen RBD varies under 300, t was set as 300 in our experiments, which could be increased if required.

The networks were implemented in Python using packages Tensorflow and Keras. Each model was end-to-end trained using Adam optimizer for 800 epochs, with a batch size of 24, and the initial learning rate set as 0.001.

Evaluation criteria using Receiver Operating Characteristics

The criteria include recall (or sensitivity, hit rate, true positive rate), selectivity (Selec. or specificity, true negative rate), precision (Prec. or positive predictive value), negative predictive value (NPV), accuracy (Acc.), and balanced accuracy (B-Acc.):

$$\text{Recall} = \frac{\text{TP}}{\text{TP} + \text{FN}}$$

$$\mathrm{Selec} = \frac{\mathrm{TN}}{\mathrm{TN} + \mathrm{FP}}$$

$$\mathrm{Prec} = \frac{\mathrm{TP}}{\mathrm{TP} + \mathrm{FP}}$$

$$\mathrm{NPV} = \frac{\mathrm{TN}}{\mathrm{TN} + \mathrm{FN}}$$

$$\mathrm{Acc} = \frac{\mathrm{TP} + \mathrm{TN}}{\mathrm{TP} + \mathrm{TN} + \mathrm{FP} + \mathrm{FN}}$$

$$\mathrm{BAcc} = \frac{\mathrm{Recall} + \mathrm{Selec}}{2}$$

Where TP, TN, FP, FN respectively denote the data sample number of true positive, true negative, false positive, false negative, with thresholding value of prediction probability p_{thresh}:

$$\mathrm{TP} = |\{(s_{\mathrm{h}}, s_{\mathrm{l}}, s_{\mathrm{a}})|p(s_{\mathrm{h}}, s_{\mathrm{l}}, s_{\mathrm{a}}) > p_{\mathrm{thresh}} \in\} \cap (\text{positive binding})|$$

$$\mathrm{TN} = |\{(s_{\mathrm{h}}, s_{\mathrm{l}}, s_{\mathrm{a}})|p(s_{\mathrm{h}}, s_{\mathrm{l}}, s_{\mathrm{a}}) \leq p_{\mathrm{thresh}} \in\} \cap (\text{negative binding})|$$

$$\mathrm{FP} = |\{(s_{\mathrm{h}}, s_{\mathrm{l}}, s_{\mathrm{a}})|p(s_{\mathrm{h}}, s_{\mathrm{l}}, s_{\mathrm{a}}) \leq p_{\mathrm{thresh}} \in\} \cap (\text{positive binding})|$$

$$\mathrm{FN} = |\{(s_{\mathrm{h}}, s_{\mathrm{l}}, s_{\mathrm{a}})|p(s_{\mathrm{h}}, s_{\mathrm{l}}, s_{\mathrm{a}}) > p_{\mathrm{thresh}} \in\} \cap (\text{negativebinding})|$$

Precision - Recall Curve (PRC) plotted by Recall and Precision with varying thresholding value of prediction probability to evaluate the models' performance with the strategies balanced between aggressively maximizing positives detection and conservatively avoiding false detection. Because the positive data (3265 binding pairs) is much smaller than the negative data (2071 non-binding pairs and 14×11444 assumed non-binding pairs) as described previously, Receiver Operating Characteristics (ROC) is also used to further illustrate the capability of binding prediction balanced between positive and negative (**Supplementary information, Fig. S1b**). ROC is a curve plotted by Recall and Selectivity with varying thresholding value of prediction probability, thus can demonstrate the capability of the binding prediction with varying condition of

thresholding based on a positive-negative imbalanced validation dataset. The prediction score of the model gives the probability of paired heavy and light chain to bind to given antigens, but not the affinity of the antibody. Therefore, there is no correlation between ELISA OD and prediction score (**Supplementary information, Fig. S1c**).

The ACNN based XBCR-net achieved a high selectivity (92%) with the validation dataset, indicating that it had a low false-positive prediction rate. Therefore, we hypothesized that XBCR-net could predict the antigen-specific BCRs from scBCR-seq data. To test the accuracy of our model in scBCR-seq data, we performed the prediction using single-cell data from the GEO database (GSE171703) that were not covered by our training and validation dataset as outlined (**Supplementary information, Data S2**).

First, the amino acid sequences of the paired VH and VL are encoded as input for XBCR-net. These sequences were checked to be in-frame and not present in the training dataset. XBCR-net then predicted the binding probability of each VH-VL pair, ranging from "0" to "1" against the RBD of SARS-CoV-2. We have taken the predicted probability greater than "0.5" as the positive binding while smaller and equal to "0.5" as the negative. We then compared our predictions with the experimentally confirmed RBD binding BCRs from the same scBCR-seq dataset (GSE171703, single cell BCR sequencing from SARS-CoV-2 wild type infected patients) as an independent validation. We have trained nine models of XBCR-net, including the models with three varying random initialization seeds and different data folder selections for training and validation, respectively, based on ACNN, CNN, and FCN. The prediction results of these 9 models were then compared with the binding information of the mAbs cloned from the same scBCR-seq data (**Supplementary information, Fig. S5**). The performance of our model is then compared with the published transformer based neural network (**Supplementary information, Table S4**)

Antibody cloning and purification

The method was described in details in previous studies. The variable region of paired heavy and light chains was synthesized and cloned into vectors expressing the constant region of human IgG1. The vectors were then transfected into 293F cells by polyetherimide (PEI) at 1 mg/ml. The supernatant was collected at day 5 and purified by protein A embedded columns. The antibody concentration was measured by nanodrop.

Antibody sequence analysis

The antibody sequences are analyzed by IMGT-high-V-quest to gain the detailed information of V gene usage, CDR and FR region sequences. The sequences are then processed with ChangeO and VDJtools as described in earlier papers. The V gene and J gene usage was plotted with the VDJtools package while the statistics were done by Prism 8.0. The CDR-H3 length and sequence dominance was also analysed and plotted by VDJtools package. To cluster the CDR-H3, antibodies with the same CDR-H3 length and more than 80% similarity were allocated to the same cluster by using a deterministic clustering approach[4]. The antibodies in different clusters, therefore should have more than 20% difference in CDR-H3. Sequence logos were generated with online python based tool. We defined pan-SARS2 as antibodies that bind to SARS-CoV-2 and its alpha, beta, delta, gamma and omicron variants.

Criteria for antibody selection from the prediction

The antibodies that received a prediction score higher than 0.5 by all three ACNN based XBCR-net models are first chosen. These antibodies were then further filtered by the best XBCR-net with a prediction score higher than 0.99. Because of the biased IGHV3-

30 usage we observed in the predicted cross-reactive RBD binders, we selected 10 IGHV3-30 antibodies in addition to the other 15 antibodies with diverse IGHV usage from the filtered antibody lists.

Anti-RBD ELISA

The NUNC 96-well plate was coated with 1 μg/ml RBD of SARS-CoV-2, SARS-CoV-2 omicron or SARS-CoV overnight at 4 °C. The plates were washed with PBST (0.1% Tween) three times before blocking with 3% BSA for one hour on the shaker. The mAbs were diluted to 1 μg/ml to co-incubate with the coated antigens for one hour before washing and subsequent co-incubation with anti-human IgG-AP (Sigma). The colour was developed by PNPP tablets (Sigma), followed by optical density at 450 nm (OD450) recording with ELISA microplate reader. An antibody is defined as ELISA-positive when the OD450 is three times larger than the negative control, which uses an H7N9-specific human IgG1 antibody (HG1K, Sino Biological).

Pseudovirus neutralization assay

The pseudovirus neutralization was described in details in previous studies[5]. The mAbs were diluted to six gradients in the total DMEM and mixed with pseudovirus solution to 96-well plate. Huh7 cells were cultured in high glucose DMEM supplemented with 10% FBS, 100 U/ml Penicillin-streptomycin, and 20 mM HEPES in 37 °C incubators. The Huh7 was trypsinized and seeded to the mAb-pseudovirus plate at 2×10^5 cells/ml. After 24 hours of incubation at 37 °C the culture supernatant was carefully aspirated to leave 100 μl in each well. The luciferase substrate was then added to the wells; then150 μl of cell lysate was transferred to the white opaque 96-

well microplate for luciferase detection with microplate luminometer. Each group of plasma/serum samples contained two replicates.

Statistical Analysis

Statistical analyses were performed and specified in the figure legend. IC_{50} was determined by a four-parameter non-linear regression using GraphPad Prism 9.0.

Materials

Reagent	Supplier	Amount used
293F cells	Thermo Fisher R790	
Huh7 cells	Thermo Fisher	
polyetherimide (PEI)	Sigma	1 mg/ml
Anti-human IgG gamma chain specific	Sigma	1:5000
Anti-human IgG Fc specific alkaline phosphatase	Sigma	1:1000
PNPP tablet	Sigma	
RBD SARS-CoV-2	Sino biological	0.2 µg/ml or 1 µg/ml
RBD SARS-CoV-2 Omicron	Sino biological	0.2 µg/ml or 1 µg/ml
RBD SARS-CoV	Sino biological	0.2 µg/ml or 1 µg/ml
Omicron Pseudovirus	Gift from Prof Xiaoliang Xie’s lab	

Delta Pseudovirus	Gift from Prof Xiaoliang Xie's lab	
HG1K antibody	Sino biological	1 µg/ml
DMEM	Gibco	
HEPES	Thermo Fisher	
Penstrip	Sigma	

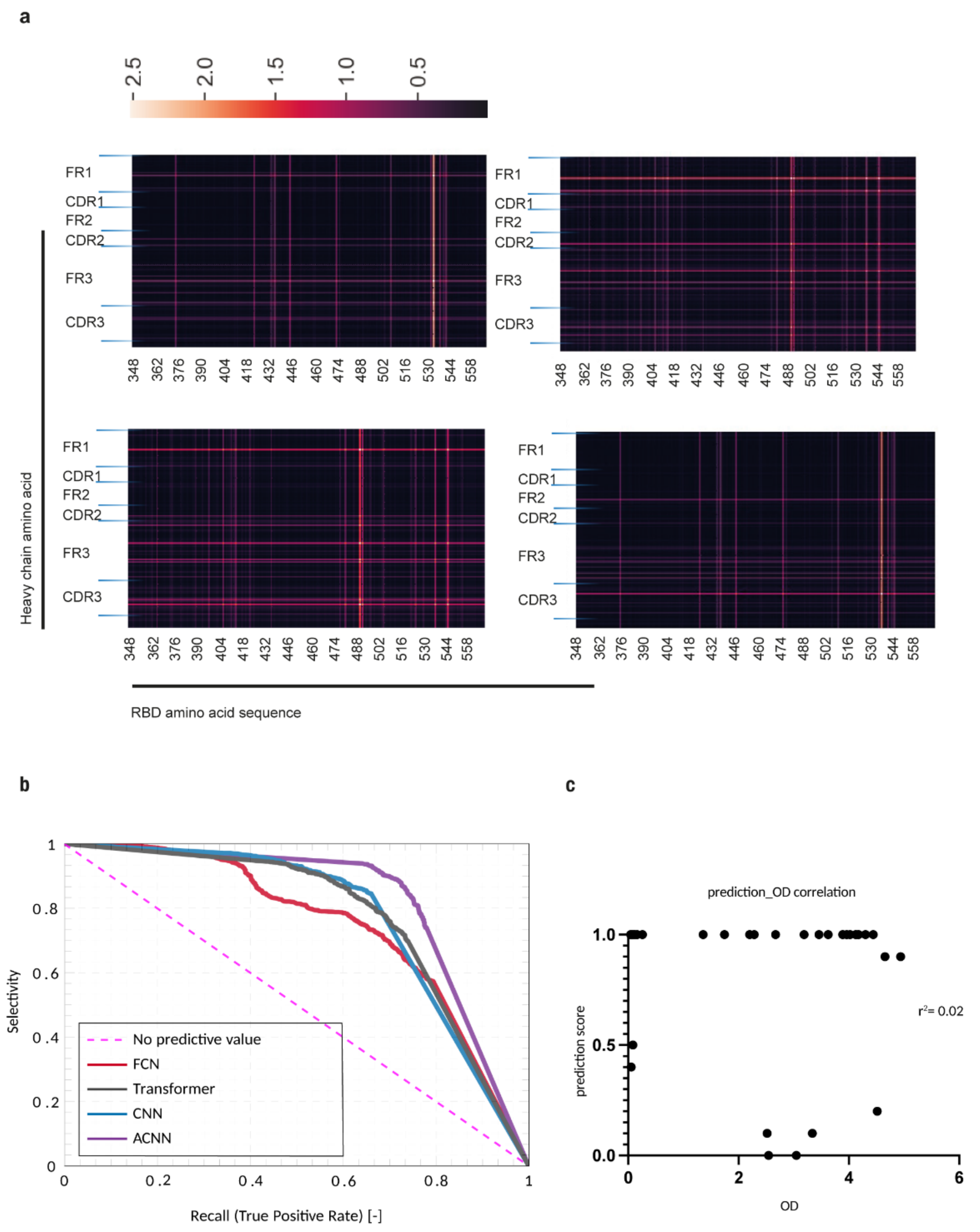

Supplementary information, Fig. S1 Impact score of antibody-antigen interaction and AUC/ROC of the three models. a Impact score of four representative cross-reactive antibodies against SARS-CoV-2 and omicron variants. **b** AUC/ROC of the Selectivity vs Sensitivity of the three models (FCN, ACNN, Transformer and CNN) on the validation dataset. The no predictive value is plotted in dotted line as negative control. **c** The correlation of OD value and the prediction score of the 25 anti-SARS-CoV-2 antibodies.

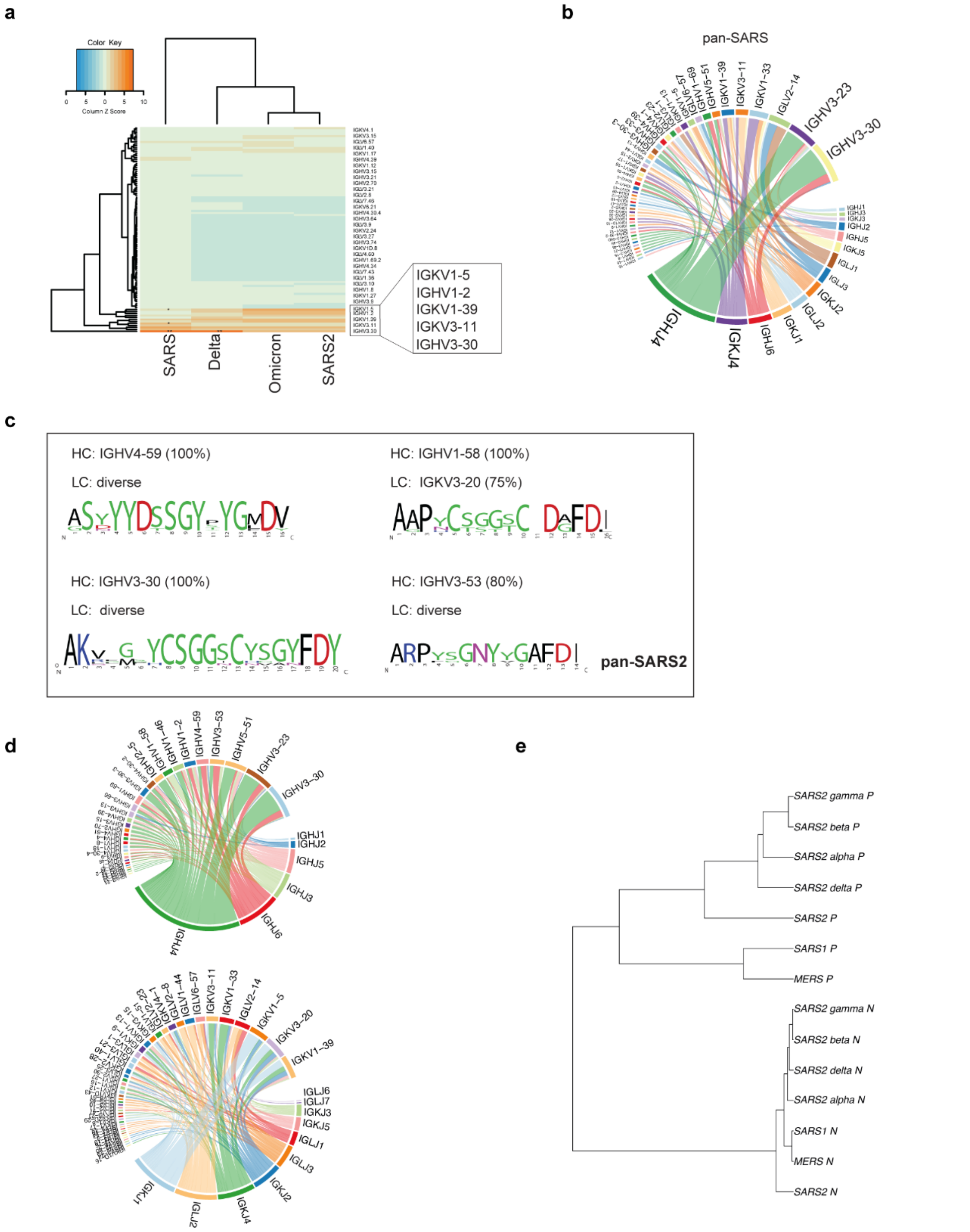

Supplementary information, Fig. S2 XBCR-net-predicted binders display biased HV3-30 usage. a Heatmap showing the difference between IGHV, KV, LV and IGHJ, KJ, LJ usage frequency of the predicted SARS-CoV-2, omicron variants, delta variant and SARS-CoV-1 binders, respectively. Color indicates the Z score from -8 (blue) to 8 (red), blue indicates low number of antibodies using the gene and red indicates high number of antibodies using the gene, Wilcoxon test FDR adjusted P value: $^{*}P < 0.05$,

$P < 0.005$. **b Circos plots showing the antibody sequences that are grouped into pan-SARS (pan-SARS2 plus reactive to SARS-CoV). The V region to J region pairing of the pan-SARS sequences is analyzed. The length of the outer ring indicates the frequency of antibodies using the gene. **c** The CDR3 sequences of the predicted pan-SARS and pan-SARS2 binders are clustered by using a 80% sequence similarity. For cluster that has size larger than 4, the CDR-H3 sequences of the heavy chain are visualized as a sequence logo plot, where y axis stands for the frequency of the individual amino acid at the corresponding position in x axis. The frequency of the dominating VH and VL gene is listed above the logo. Diverse means none of the VL gene account for more than 40% of the total antibodies in the cluster. **d** Circos plot of the heavy chain and light chain of the antibody sequences that are grouped into pan-SARS-2. **e** The hierarchical analysis of the predicted SARS-CoV (SARS1 P), SARS-CoV-2 (SARS2 P) and SARS-CoV-2 VOCs P, MERS-CoV (MERS P) binders. The antibodies that do not bind to SARS-CoV (SARS1 N) and other Ns are also analyzed.

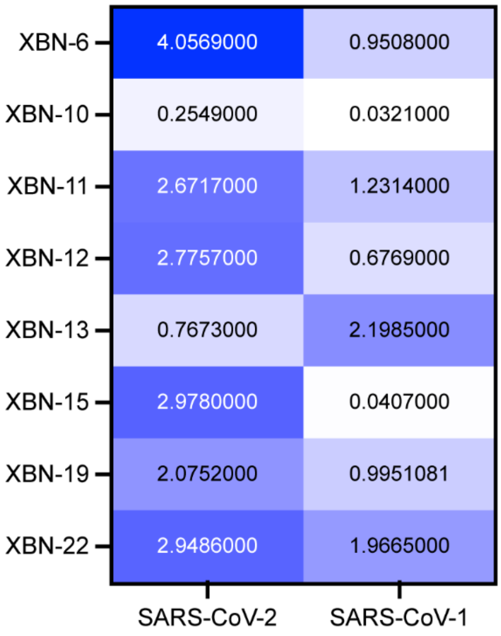

Supplementary information, Fig. S3 Heatmap showing antibodies binding to SARS-CoV in addition to SARS-CoV-2 by ELISA. The binding of predicted SARS-CoV binders at 1 µg/ml to SARS-CoV and SARS-CoV-2 was detected by ELISA, with a cut-off value of OD = 0.1. $n = 3$. Representative data is shown.

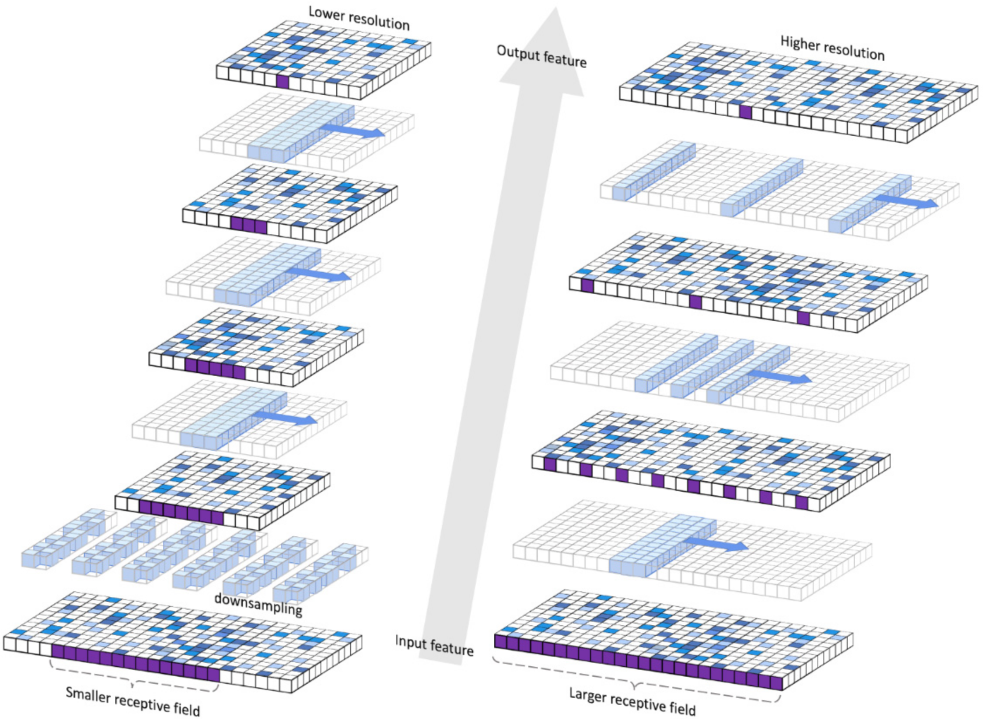

Supplementary information, Fig. S4 Construction of Atrous-III blocks.

Compared with Res-conv block, Atrous-III block utilizes the convolution with atrous rates computationally optimized to extend the receptive field without any losses of resolution resulting from down-sampling. Thus, Atrous-III block is able to achieve broader field of view with higher resolution than Res-conv block as shown in the figure below, providing more comprehensive and informative features.

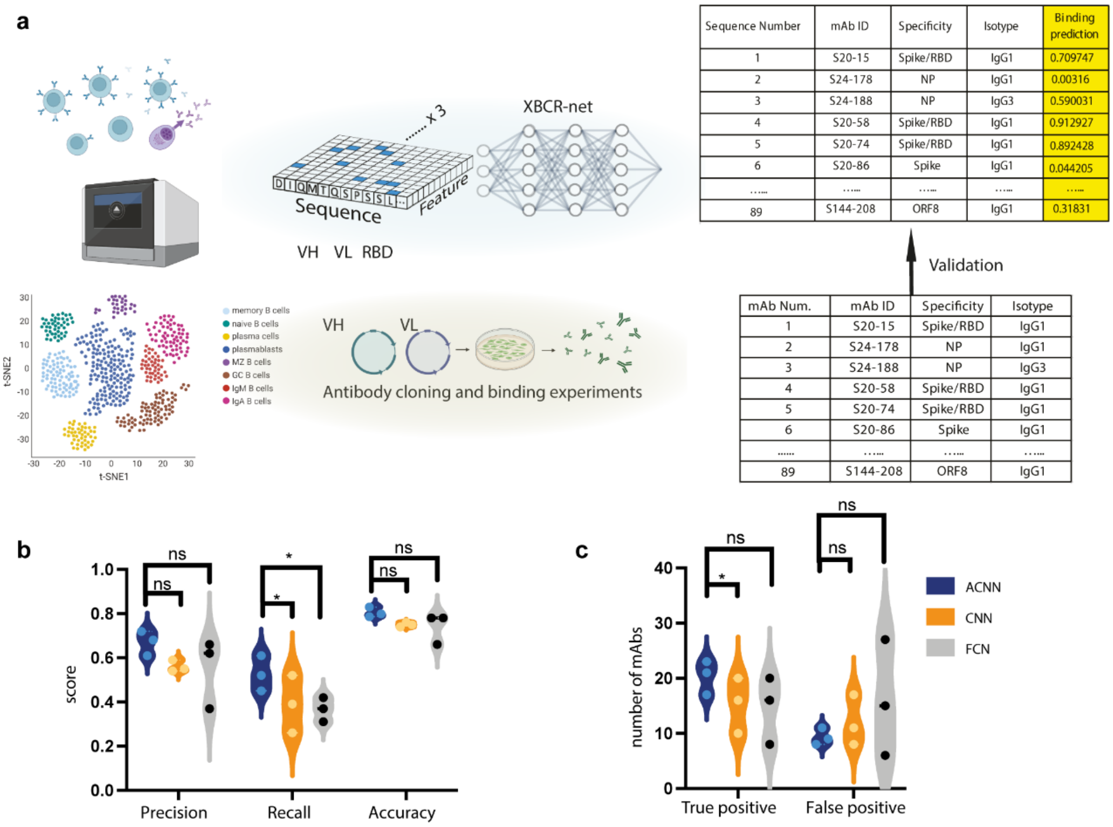

Supplementary information, Fig. S5 ACNN-based XBCR-net can be applied to identify anti-SARS-CoV-2 antibodies from single-cell BCR data. a Independent validation of the XBCR-net on BCR single-cell seq data. The paired heavy and light variable sequences (total 15600 amino acid sequences) were extracted from the single-cell BCR data as input for the prediction. The XBCR - net prediction was then compared with the experimental data from the 89 randomly cloned monoclonal antibodies against SARS-CoV-2 RBD region. **b** Based on the binding affinity of the 89 mAbs to the RBD of SARS-CoV-2, they are grouped into binding and non-binding groups. The binding prediction of the 89 mAbs to RBD by XBCR-net, CNN and FCN was then compared with the experimental data to calculate the precision, recall and accuracy metrics. Three networks were trained for each construction and the scores are analysed. N=3 models for each construction T test adjusted P value: $*P < 0.05$, $**P < 0.005$. **c** The true positive and false positive number of predictions of XBCR-net, CNN and FCN are compared. N=3 models.

Supplementary information, Table S1 ACNN-based XBCR-net can predict SARS-CoV-2 omicron variant binding.

Evaluation through accuracy, recall (also known as sensitivity), selectivity (Sele.), positive and negative predictive value (PPV/Precision and NPV), and balanced-accuracy (B-Acc.).

Models	Acc	Recall	Sele.	Precision	NPV	B-Acc.
ACNN-based XBCR-net	77%	72%	82%	80%	74%	77%

Supplementary information, Table S2 The IC_{50} of the mAb neutralization against delta and omicron variant pseudovirus.

mab ID	Delta IC_{50} (μg/ml)	Omicron IC_{50} (μg/ml)	Cross-reactivity		
			WT	Omicron	SARS-CoV
XBN-1	0.007	0.42	Binding	Binding	-
XBN-3	Inf	Inf	Binding	Binding	-
XBN-6	1.28	Inf	Binding	Binding	Binding
XBN-10	Inf	Inf	Binding	Binding	-
XBN-11	Inf	0.017	Binding	Binding	Binding
XBN-12	Inf	Inf	Binding	Binding	-
XBN-19	Inf	Inf	Binding	Binding	Binding
XBN-22	Inf	Inf	Binding	Binding	Binding
XBN-23	Inf	Inf	Binding	Binding	-
XBN-24	Inf	Inf	Binding	Binding	-

Supplementary information, Table S3 Five folder cross validation of FCN, CNN, Transformer and ACNN based XBCR-net (Supplementary methods: neural network).

Model		Folder	Evaluation (threshold possibility = 50%)				
Framework	1st part		Accuracy	Recall	Precision	Selectivity	NPV
XBCR-net	FCN	1	46.2%	99.9%	46.2%	0.0%	50.0%
	Transformer		68.4%	62.7%	63.1%	72.6%	72.3%
	CNN		65.6%	50.0%	67.2%	79.0%	64.8%
	ACNN		76.5%	61.6%	83.2%	89.3%	73.0%
	FCN	2	75.0%	42.0%	59.2%	88.3%	79.1%
	Transformer		77.7%	36.6%	72.1%	94.3%	78.7%
	CNN		77.1%	23.8%	86.4%	98.5%	76.2%
	ACNN		78.5%	96.3%	57.4%	71.3%	97.9%
	FCN	3	75.1%	62.0%	77.2%	85.4%	73.8%
	Transformer		77.6%	64.8%	80.8%	87.7%	75.8%
	CNN		78.5%	65.1%	82.6%	89.1%	76.2%
	ACNN		79.6%	68.0%	83.0%	88.9%	77.7%
	FCN	4	75.2%	19.7%	75.7%	97.5%	75.1%
	Transformer		79.0%	39.6%	75.2%	94.8%	79.6%
	CNN		79.3%	39.4%	77.4%	93.7%	79.7%
	ACNN		82.4%	59.1%	74.3%	91.8%	84.8%
	FCN	5	78.7%	85.6%	73.6%	72.4%	84.9%
	Transformer		83.6%	69.3%	94.8%	96.5%	77.8%
	CNN		83.5%	68.9%	94.8%	96.6%	77.6%
	ACNN		84.9%	70.1%	97.4%	98.3%	78.5%
	FCN	Avg.	70.0%	61.8%	66.4%	68.7%	72.6%
	Transformer		77.3%	54.6%	77.2%	89.2%	76.8%
	CNN		76.8%	49.4%	81.7%	91.4%	74.9%
	ACNN		80.4%	71.0%	80.1%	87.9%	82.4%

Supplementary information, Table S4 The performance of XBCR-net compared with transformer based network.

	Accuracy	Recall	Precision	Selectivity
XBCR-net	80.4%	71.0%	80.1%	87.9%
Transformer[4] (Immunity 2022)	65.8%	35.3%	82.9%	93.4%

References

1. Zhou, X. Y., Zheng, J. Q., Li, P. & Yang, G. Z. in *2020 IEEE International Conference on Robotics and Automation (ICRA)* 8455-8461 (2020).
2. He, K., Zhang, X., Ren, S. & Sun, J. in *Proceedings of the IEEE Conference on Computer Vision and Pattern Recognition* 770-778 (2016).
3. Raybould, M. I. J., Kovaltsuk, A., Marks, C. & Deane, C. M. *Bioinformatics* **37**, 734-735 (2021).

4 Wang, Y. *et al. Immunity* **55**, 1105-1117.e4 (2022).

5. Cao, Y. *et al. Nature* **602**, 657-663 (2022).

Supplementary information, Data S1. (separate file)

Training and validation data for the XBCR-net. The data contains (i): 3265 anti-RBD antibodies against 14 variants of RBD sequences, (ii): 2071 antibodies that do not bind RBD, (iii): 5066 anti-HIV antibodies, (iv): 6378 anti-HCV antibodies, (v): anti-RSV, anti-Ebola, anti-flu and baseline antibodies. Full antibody heavy chain and light chain amino acids are encoded as the **input I, II**. Amino acid sequences of RBD of 14 variants are encoded as **input III**.

Validation dataset contains 142 anti-omicron antibodies that are derived from 5 different published studies (Zijun Wang et al, 2022; Wanwisa Dejnirattisai et al, 2022; Rungtiwa Nutalai et al, 2022; Kang Wang et al, 2022; Davide Robbiani et al, 2020). They bind to omicron BA.1. The other 142 anti-SARS-CoV-2 antibodies do not bind to omicron variant BA.1, which were used as negative sample.

Supplementary information, Data S2. (separate file)

Amino acid sequences from the single cell BCR data for the XBCR-net prediction.

Supplementary information, Data S3. (separate file)

Prediction score of the therapeutic antibodies against new omicron variants BA.1 BA.2, BA.4 and BA.5.
